# Effect of Phlorotannins from Brown Algae *Costaria costata* on α-*N*-Acetylgalactosaminidase Produced by Duodenal Adenocarcinoma and Melanoma Cells

**DOI:** 10.3390/md21010033

**Published:** 2022-12-30

**Authors:** Irina Bakunina, Tatiana Imbs, Galina Likhatskaya, Valeria Grigorchuk, Anastasya Zueva, Olesya Malyarenko, Svetlana Ermakova

**Affiliations:** 1 G.B. Elyakov Pacific Institute of Bioorganic Chemistry, Far Eastern Branch, Russian Academy of Sciences, 159 Pr-t 100-let Vladivostoka Str., 690022 Vladivostok, Russia; 2 Federal Scientific Center of the East Asia Terrestrial Biodiversity, Far Eastern Branch, Russian Academy of Sciences, 159 Pr-t 100-let Vladivostoka Str., 690022 Vladivostok, Russia

**Keywords:** α-N-acetylgalactosaminidase, α-NaGalase, inhibitor, phlorotannins, brown algae, *Costaria costata*, carcinoma, melanoma, cancer cells

## Abstract

The inhibitor of human α-N-acetylgalactosaminidase (α-NaGalase) was isolated from a water–ethanol extract of the brown algae *Costaria costata*. Currently, tumor α-NaGalase is considered to be a therapeutic target in the treatment of cancer. According to NMR spectroscopy and mass spectrometric analysis, it is a high-molecular-weight fraction of phlorethols with a degree of polymerization (DP) equaling 11–23 phloroglucinols (CcPh). It was shown that CcPh is a direct inhibitor of α-NaGalases isolated from HuTu 80 and SK-MEL-28 cells (IC_50_ 0.14 ± 0.008 and 0.12 ± 0.004 mg/mL, respectively) and reduces the activity of this enzyme in HuTu 80 and SK-MEL-28 cells up to 50% at concentrations of 15.2 ± 9.5 and 5.7 ± 1.6 μg/mL, respectively. Molecular docking of the putative DP-15 oligophlorethol (P15OPh) and heptaphlorethol (PHPh) with human α-NaGalase (PDB ID 4DO4) showed that this compound forms a complex and interacts directly with the Asp 156 and Asp 217 catalytic residues of the enzyme in question. Thus, brown algae phlorethol CcPh is an effective marine-based natural inhibitor of the α-NaGalase of cancer cells and, therefore, has high therapeutic potential.

## 1. Introduction

α-N-Acetylgalactosaminidase (α-NaGalase) (EC 3.2.1.49) removes α-linked residues of N-acetylgalactosaminide from the non-reducing ends of various complex carbohydrates and glycoconjugates. Glycolipids, glycopeptides, and glycoproteins; blood group A erythrocyte antigens [1,2,3]; lipopolysaccharides of the cell walls; and capsules of bacteria [4,5,6] are physiological substrates for α-NaGalase. This enzyme is widespread in the organs and tissues of mammals, bacteria, and fungi.

Human α-NaGalase is a lysosomal enzyme encoded by the sole NAGA gene localized to chromosome 22q13→qter [7]. This enzyme’s structure, mechanism of action, and role in the human body have been studied in detail [8,9]. According to the structural classification of carbohydrate-active enzymes (CAZy), this enzyme belongs to the 27 family of glycoside hydrolases (GH27) [10]. It is produced by all cancer cells and accumulates in the blood plasma of cancer patients [11]. This enzyme, which is released from cancer cells, is active at pH 6.0–6.8 and has endo-type activity, hydrolyzing the O-glycosidic linkage between α-N-acetylgalactosamine and serine or threonine in glycoproteins [12]. These properties of the enzyme prevent macrophage activation in cancer patients via the deglycosylation of the vitamin D_3_-binding protein, which is the precursor of the macrophage-activating factor (GcMAF) [13,14]. The level of enzyme activity and number of its forms increase in blood serum, especially at the initial stage of the disease and the stage of metastasis [15]. A high level of the enzyme activity leads to immunosuppression in patients with advanced cancer [16]. Thus, α-NaGalase, as an immunosuppressive agent in cancer patients, is considered to be a potential therapeutic target in cancer treatment.

The activity of this aggressive enzyme can be suppressed in various ways. Knockdown of the NAGA gene with a transfection reagent in EPG85.257RDB cells leads to an increase in the rate of late apoptosis and to augmentative and regressive effects on cell death and migration [17]. A study of the effects of Naga-shRNA downregulation in the MCF-7 (human breast carcinoma) and A2780 (human ovarian carcinoma) cell lines showed significant inhibition of the migratory and invasive properties of cancer cell lines [18]. However, the epigenetic modification of the NAGA gene by DNA hypermethylation reduced the expression of α-NaGalase and increased chemoresistance to cisplatin in ovarian cancer [19]. Previously, we have shown that the treatment of DLD-1 adenocarcinoma cells with fucoidan from the brown alga *Fucus evanescens* reduces the production of this enzyme [20]. Some aaptamines and makaluvamines isolated from marine sponges showed no direct inhibitory effect on cancer-associated α-NaGalase; however, isoaaptamine, 9-demethylaaptamine, damirone B, and makaluvamine H reduced the expression of the enzyme in the human colorectal adenocarcinoma cell line DLD-1 at a concentration 5 µM [21].

The polyphenolic compounds of brown algae constitute a class of oligomeric and polymeric phlorotannins. They consist of phloroglucinol (1,3,5-trihydroxybenzene) as a basic unit linked in different ways. The increased interest in these compounds over the last few years has arisen due to their wide range of biological activities. A number of reviews have summarized the results of comprehensive studies of phlorotannins in relation to their biological significance, isolation, structural features, and action, for which the latter mainly comprises antioxidant, antitumor, antidiabetic, and anti-inflammatory activities [22,23,24,25]. An especially interesting finding was that eckol stimulated the innate and adaptive immune responses responsive to tumor surveillance in mice with a sarcoma activating the phagocytic system [26]. We previously showed that the impurity of phenols plays a key role in the antioxidant activity exhibited by fucoidan from the brown alga *F. evanescens* [27]. The polyphenolic impurities contained in fucoidan fractions of brown algae *F. evanescens* reduced the protection of sea urchin embryogenesis and caused the appearance of a large number of embryos with morphological abnormalities [28]. Previous studies have described the direct inhibitory effects of phlorotannins on hyaluronidase [29], lipoxygenase [30], the glycosidases of marine mollusks [31], amylase, lipase and trypsin [32], reverse transcriptase, protease [33], and the central nervous system-related enzymes acetyl- and butyryl-cholinesterases, monoaminoxidases, β-secretase, and tyrosinase [34].

This article aimed to study the effects of phlorotannins from the brown algae *C. costata* on α-NaGalase produced by duodenal adenocarcinoma HuTu 80 and melanoma SK-MEL-28 cell lines, namely, the suppression or increase in the activity of α-NaGalase in cancer cells, as well as the direct inhibition or activation of isolated enzymes.

## 2. Results

### 2.1. Biochemical and Catalytic Properties of α-NaGalases

#### Isolation and Purification of α-NaGalase from Cell Lysates

The protein fractions enriched with α-NaGalase activity were isolated from the cell lysates of duodenal adenocarcinoma HuTu 80 and melanoma SK-MEL-28 cell lines in accordance with procedures described previously [20,21]. The following study of the biochemical characteristics of α-NaGalase is necessary for the investigation of phlorotannins’ effects on the enzyme’s activity.

**Figure 1:**
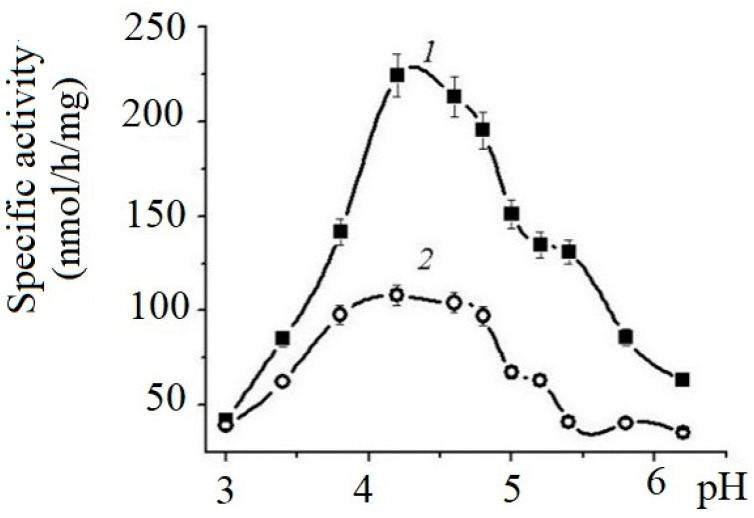
Effects of pH on the specific activity of α-NaGalases from HuTu 80 (1) and SK-MEL-28 (2) cell lines. Solution of 0.05 M sodium citrate buffer.

The effects of pH on the enzyme’s activity are shown on Figure 1.

The enzymes of both cell lines exhibit maximum activity in the pH range from 4.0 to 5.5. The specific activity of HuTu 80 α-NaGalase exceeded the activity of the SK-MEL-28 enzyme by almost threefold. 

To determine the Michaelis–Menten constant (*K*_m_) and the maximum reaction rate (*V*_max_) of the α-NaGalase of both cell lines, the concentration of the pNPNAGal substrate was varied from 0.07 to 9.0 mM in a solution of Na citrate with pH 4.5.

This experiment showed that the *K*_m_ values of the HuTu 80 and SK-MEL-28 enzymes differ slightly, but the *V*_max_ value of HuTu 80 α-NaGalase was almost three times higher than the *V*_max_ value of the SK-MEL-28 enzyme (Table 1). The high *K*_m_ values and low *V*_max_ values of the studied enzymes probably characterize their low affinity for the commercial chromogenic standard substrate.

### 2.2. Phlorotannins of Brown Algae C. costata

#### 2.2.1. Brown Alga Collection and Phlorotannins’ Isolation

The fraction of phlorotannins (CcPh) was isolated from the water–ethanol extract of the brown algae *C. costata* using sequential liquid extraction with organic solvents and chromatography on silica gel, polychrome-1, and C-18 as described earlier with some modifications [35]. The CcPh fraction was characterized by nuclear magnetic resonance (NMR) spectroscopy and mass spectrometry, as described below.

#### 2.2.2. Nuclear Magnetic Resonance Analysis

**Figure 2:**
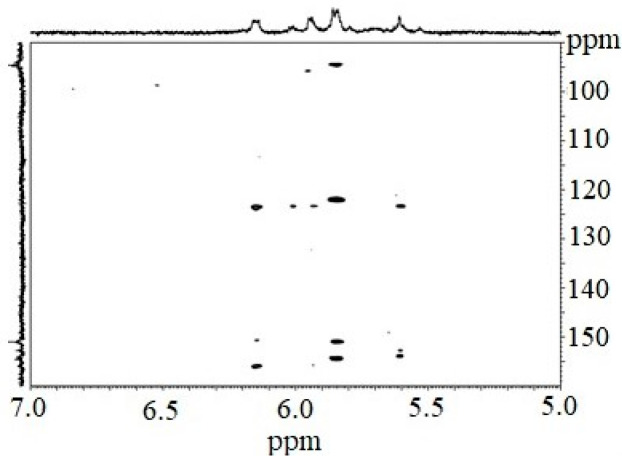
Fragmented HMBC spectrum of the CcPh fraction.

The ^1^H NMR spectrum of the CcPh fraction showed a distribution of ^1^H signals between 5.8 and 6.3 ppm, which is typical for polyphenols. Observations in experiments with HMBC (Figure 2, Table 2) confirmed the presence of phlorotannin structures.

The HMBC spectrum showed characteristic carbon atom resonances at 93.8–94.5 ppm corresponding to unsubstituted benzene carbons; signals in the range of 122.0 to 123.6 ppm corresponded to diaryl–ether bonds (phloroglucinol units connected by a simple ether); and signals between 150.9 and 156.0 ppm were characteristic of benzene carbon-bearing hydroxylated groups.

However, signals at 100–105 ppm and 142–148 ppm were not present. This fact indicates the absence of aryl–aryl carbon atoms, as well as the presence of additional OH functions other than 1,3,5-OH groups. Thus, fucolic type units and fugalol type units are absent in the studied sample. It can be concluded that this polymer belongs to the phlorethol class of the phlorotannins.

#### 2.2.3. Mass Spectra Analysis

**Figure 3:**
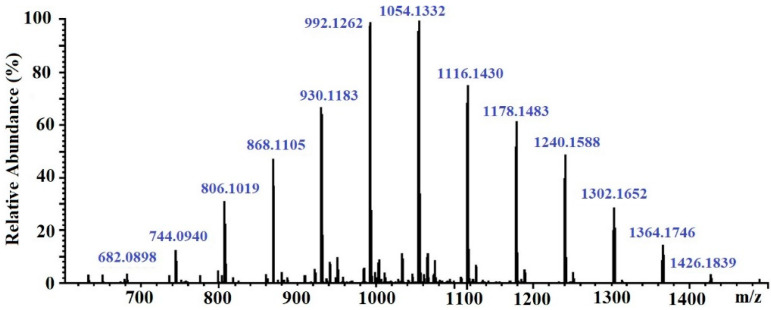
ESI–MS spectra, operating in negative mode (ES-), of phlorethols fraction CcPh from *Costaria costata*.

The molecular weight of the CcPh fraction was determined by ESI–MS. Negative ESI–MS measurements showed the mixture of pseudomolecule ions of CcPh at *m*/*z* 744 to *m*/*z* 1364 [M − 2H]^−2^. The degree of phloroglucinol polymerization ranged from 12 to 22 phloroglucinol units in the CcPh fraction, with the most abundant phlorethols containing between 15 and 18 phloroglucinol units (*m*/*z* 930 to *m*/*z* 1116 [M – 2H]^−2^) (Table 3, Figure 3)

### 2.3. The Effect of the Phlorethol CcPh on α-NaGalases in Cancer Cells

#### 2.3.1. Cytotoxic Effect of CcPh fraction on Human Duodenal Carcinoma HuTu 80 and Melanoma SK-MEL-28 Cells

For the first step, the effects of non-toxic CcPh concentrations on the viability of human HuTu 80 and SK-MEL-28 cells were tested by an MTS assay. It was shown that the concentrations of CcPh that caused a 50% reduction in cell viability (*IC*_50_) were 92 ± 3 µg/mL and 102 ± 5 µg/mL for the HuTu 80 and SK-MEL-28 cells, respectively. Phlorethol CcPh was non-cytotoxic up to 40 µg/ml for both cell lines.

#### 2.3.2. The Inhibitory Potency of the CcPh for α-NaGalases in Cancer Cells

**Figure 4:**
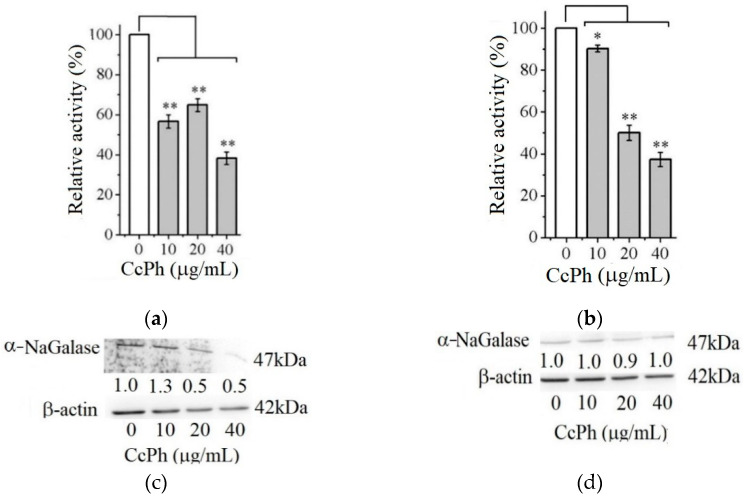
Relative activity of α-NaGalase of lysates after treatment of duodenal cancer cells HuTu 80 (**a**) and melanoma SK-MEL-28 (**b**) with 10, 20, and 40 μg/mL of the CcPh compared to the enzyme of untreated cells as positive control (0). Relative activity of α-NaGalase was defined as *A*/*A*_0_ × 100 (%), where *A* and *A*_0_ are the specific activities of α-NaGalase of the samples and the control experiment (0), respectively. The results of the α-NaGalase protein expression assay conducted via Western blot analysis are as follows: α-NaGalase protein expression was significantly decreased by CcPh compared to controls for HuTu 80 (**c**) and SK-MEL-28 (**d**). Data are shown as means ± standard deviation (SD) of values from three independent experiments. Student’s *t*-test was used to evaluate the data with the following significance levels: * *p* < 0.05 and ** *p* < 0.01. Corresponding signal intensities were determined in a densitometrical manner and normalized to total protein (β-actin) in each lane and are given below for each data point.

Figure 4 shows the effect of the phlorethol CcPh on the production of the enzyme α-NaGalase by the HuTu 80 and SK-MEL-28 cancer cells.

As can be seen from Figure 4, the activity of the α-NaGalases in the lysates of the treated cancer cells of both cancer lines decreases with an increase in the CcPh concentration up to 40 µg/mL (Figure 4a,b). The CcPh fraction reduces α-NaGalase activity by 50% in the HuTu 80 and SK-MEL-28 cells at concentrations (*IC*_50_) of 15.2 ± 9.5 and 5.7 ± 1.6 µg/mL, respectively. However, it should be noted that this compound is an inhibitor of enzyme biosynthesis only in the HuTu 80 cancer cells (Figure 4c) and not in the SK-MEL-28 cancer cells (Figure 4d). According to the results of the Western blotting analysis, the level of α-NaGalase protein after the dose-dependent treatment with CcPh decreased in the HuTu 80 cells (Figure 4c) but did not change in the SK-MEL-28 cells (Figure 4d).

### 2.4. The Phlorethol CcPh as Direct Inhibitors of α-NaGalases

**Figure 5:**
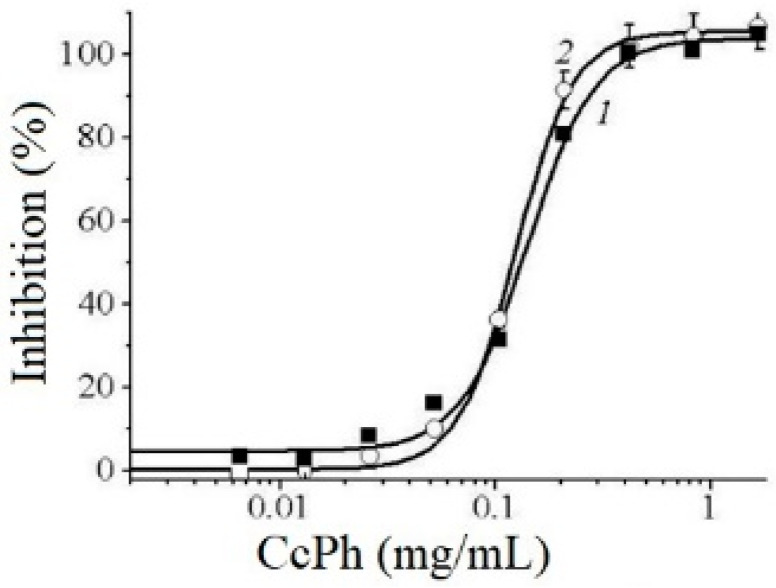
Dose–response curves of HuTu 80- (1) and SK-MEL-28-related (2) α-NaGalases inhibition after preincubation with CcPh for 30 min, followed by addition of substrate and 4 h incubation with enzyme and substrate. Inhibition (%) are plotted against concentration of CcPh on a logarithmic scale.

The potency of CcPh as a direct inhibitor was studied by a standard end-point assay. Figure 5 shows the dose–response curves regarding the inhibition of HuTu 80 α-NaGalase (1) and SK-MEL-28 α-NaGalase (2) by CcPh at final concentrations from 0 to 1.67 mg/mL. The *IC*_50_ values evaluated from the coefficients of the sigmoid curves with nonlinear regressions were 0.14 ± 0.008 and 0.12 ± 0.004 mg/mL.

To determine the reversibility of the action of CcPh action towards the α-NaGalase activity, we carried out the dialysis of the inactivated enzymes. The activity of the enzymes did not recover after dialysis against the buffer solution for 60 h, but the enzyme in the absence of the inhibitor retained 100% activity during this dialysis process. However, the time-dependence of the *IC*_50_ values was not observed after the preincubation of CcPh with these enzymes for 5, 20, 60, and 120 min (data not shown). Thus, it was shown that CcPh is a fast-binding, irreversible inhibitor of α-NaGalase.

### 2.5. Theoretical Models of Human α-NaGalase Complexes with Oligophlorethols

#### 2.5.1. Theoretical Models of Putative Heptaphlorethol

**Figure 6:**
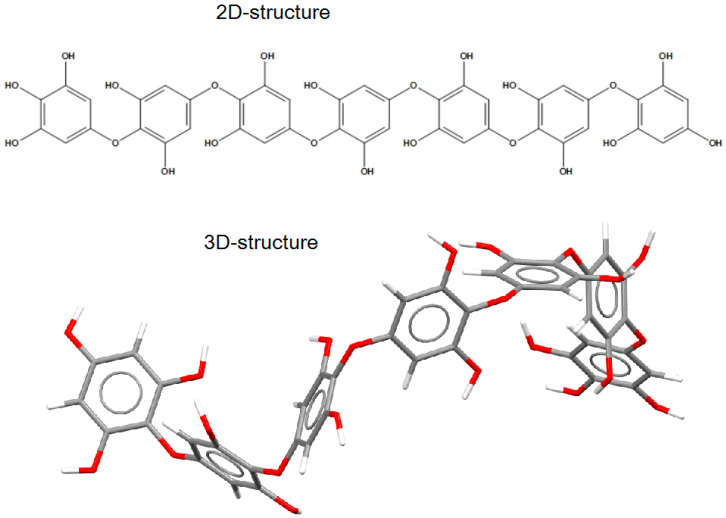
The 2D- and 3D-structures of PHPh (top and bottom picture, respectively) of putative linear oligomer consisting of seven monomeric units (phloroglucinol) linked by aryl–ether bonds.

The putative structure of the linear oligomer, termed heptaphlorethol (PHPh), which consists of seven monomeric units (phloroglucinol) linked by aryl–ether bonds, was generated using the Molecule Build module of the MOE 2020.09 program. The 2D-structure of PHPh and its optimized 3D-structure are shown on Figure 6.

#### 2.5.2. Theoretical Model of the Putative Oligophlorethol Complexes with Human α-NaGalase

**Figure 7:**
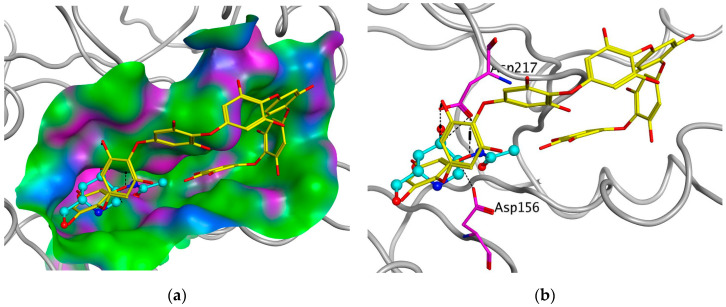
Molecular docking of human lysosomal α-NaGalase (PDB ID 4DO4) with PHPh. (**a**) Molecular surface of α-NaGalase binding site is shown in green (hydrophobic), pink (H-Bonding), and blue (mildly Polar). PHPh is shown as sticks (yellow) and iminosugar 2-acetamido-1,2-dideoxy-D-galactonojirimycin (DGNJAc) is shown as a ball-and-stick diagram (turquoise). Oxygen atoms are shown in red. (**b**) Localization of the inhibitor DGNJAc and heptaphlorethol in the active site of the human lysosomal α-NaGalase. Catalytic residues Asp 156 and 217 are shown in pink. The structure of α-NaGalase is shown as a backbond in gray.

**Figure 8:**
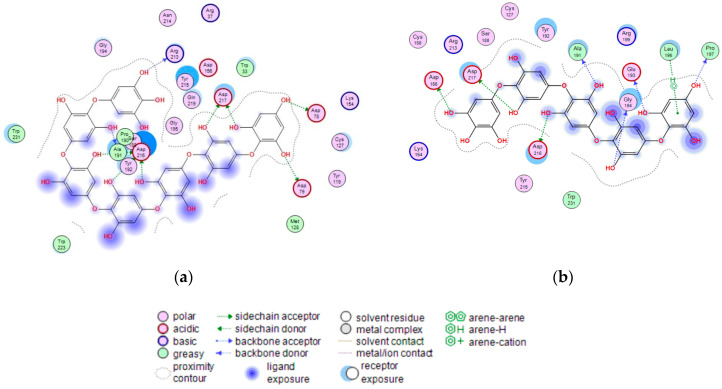
2D-diagram of contacts of the complexes of human lysosomal α-NaGalase (PDB ID 4DO4) with PHPh (**a**), and fragment of P15OPh (**b**).

To support the active-site-directed nature of α-NaGalase inactivation and assess the possible binding sites for oligophlorethols, molecular docking was performed on α-NaGalase’s active center. The active center of the α-NaGalase is located in the central (β/α)_8_ domain, in which Asp 156 and Asp217 are the catalytic nucleophile and acid/base residues, respectively. The molecular docking of the putative PHPh with α-NaGalase showed that the compound formed a complex with the active site of the enzyme. The key interactions of this inhibitor with the active site of the α-NaGalase are shown in Figure 7 and Figure 8. The molecular docking of PHPh with α-NaGalase showed that the compound forms a complex with the active site of the enzyme. The flexible PHPh molecule fills the pit around the active site’s pocket by forcefully adhering to the surface of the protein.

The terminal phloroglucinol residue enters the active site and occupies the catalytic site between Asp 156 and Asp 217. This can be seen from the superposition of α-NaGalase complexes with PHPh and the potent inhibitor of the enzyme 2-acetamido-1,2-dideoxy-D-galactonojirimycin (DGJNAc) (Figure 7a,b). The complex of the PHPh withα-NaGalase’s active center is stabilized by hydrogen bonds of hydroxyl groups with polar, acidic sidechains and basic backbones (Figure 8a). As can be seen from the superposition of the structures of the complexes PHPh and DGNJAc with the active center of the enzyme (Figure 7), the binding sites of the PHPh and DGNJAc overlap. This indicates the active-site-directed nature of the α-NaGalase’s inactivation by the compound. Furthermore, PHPh directly interacts with catalytic residue Asp 217, and its terminal phloroglucinol residue locat-ed deep in the active center blocks the substrate’s entrance into the pocket of the active center towards the catalytic residues Asp 156 and Asp 217 (Figure 7a,b).

The molecular docking of the P15OPh with α-NaGalase showed that only five monomer units of the compound form complexes with the active site of the enzyme. The remaining section of the molecule is located on the surface of the protein globule outside the active center. The model of the 3D structure of the complex of this compound with α-NaGalase is not shown. However, a 2D-diagram of the key interactions of the fragment consisting of five monomer units with the active site of α-NaGalase is shown in Figure 8b. The fragment did not present better interaction than DGNJAc, but it is also located in the active site of the enzyme and forms hydrogen bonds with catalytic residues (Table 4).

## 3. Discussion

Elevated levels of the α-NaGalase enzyme are a well-known feature of cancer cells; moreover, the ability to metastasize, disrupt programmed cell death, and exhibit drug resistance are the most obvious features of cancer cells. Currently, data on the correlation of the activity or expression of α-NaGalase genes responsible for carcinogenesis are beginning to appear in the literature. A study of the effects of Naga-shRNA suppression on the human breast carcinoma cell line MCF-7 and the human ovarian carcinoma cell line A2780 showed significant inhibition of the migratory and invasive properties of cancer cells [18]. Jafari et al. also found that after silencing α-NaGalase in the cells of human gastric adenocarcinoma, α-NaGalase downregulation had augmentative and regressive effects on cell death and migration, but no significant difference in daunorubicin resistance was observed [17]. In this paper, we studied the effects of brown algae-isolated high-molecular-weight phlorethols CcPh on the regulation of cancer-associated α-NaGalase activity.

It is known that brown algae synthesize several classes of phlorotannins, which differ with respect to the types of linkages between their phloroglucinol units. Fucols and phlorethols only consist of aryl–aryl or aryl–ether bonds, respectively, whereas fucophlorethols contain both types of linkages. Fuhalols are composed of ether-linked phloroglucinol units, with some of them containing additional hydroxyl groups other than the 1,3,5 OH functions originally present on the phloroglucinol moiety. Eckols are characterized by the presence of dibenzodioxin elements with structures of the benzofuran type in some cases [36]. It should be noted that phlorotannins have a large mass range, namely, from 126 Da to >100 kDa [36]. The molecular weight of the CcPh was determined by ESI–MS in the negative ion mode and consisted of a mixture of pseudomolecule ions at *m*/*z* 744 to *m*/*z* 1364 [M − 2H]^−2^. The most abundant phlorethols contained between 15 and 18 phloroglucinol units (*m*/*z* 930 to *m*/*z* 1116 [M − 2H]^−2^) (Table 3). Doubly charged masses are the main components in this spectrum. According to Melanson et al., with an increasing phlorotannin size, there is a greater probability of the appearance of multiple charge sites. Larger phlorotannins can be detected, as they appear as multiply charged ions under electrospray ionization conditions, gaining one negative charge for every ionized hydroxyl group (loss of H) [37]. Our mass-spectrometric analysis shows that the CcPh fraction is represented by phlorethols from 11 to 23 degrees of the polymerization of phloroglucinol, differing by one monomeric unit (Figure 3, Table 3). At present, the separation of long, condensed phlorotannins is a difficult task and is complicated by an increase in the number of isomers with an increasing polymer length. 

Previously, it was shown that phlorethols CcPh (Mw_m_ = 2520 Da) isolated from brown algae C. costata at non-toxic concentrations inhibited the human colorectal cells HCT-116 and HT-29’s colony formation ability in vitro and significantly enhanced their sensitivity to low, non-toxic X-ray irradiation [38]. The same fraction of CcPh irreversibly inactivated the recombinant enzyme endo-α-1,4-l-fucanase (EC 3.2.1.212) from the marine bacterium *Formosa alga* KMM 3553^T^ (*IC*_50_ = 39 μg/mL) [35]. Another fraction of phlorotannins from the brown algae *F. evanescens* inhibited recombinant endo-α-1,4-L-fucanase (*IC*_50_ = 22 μg/mL) [35] and α-l-fucosidase (*IC*_50_ = 29 μg/mL) from the marine bacterium *Formosa alga* KMM 3553^T^, as well as a native endo-fucanase (in which the interval of inhibiting concentrations was 30–80 μg/mL) from the marine mollusk *Patinopecten yessoensis* and β-glucosidase (*IC*_50_ = 100 μg/mL) from the marine mollusk *Littorina sitkata* [39].

To study the effect of CcPh on the activity of α-NaGalase, we chose the human duodenal adenocarcinoma HuTu 80 and melanoma SK-MEL-28 cell lines and used two assays: the first was the treatment of cells with the CcPh fraction (in vitro), while the second was the direct treatment of α-NaGalase isolated from cell lysates with the CcPh fraction. Consequently, we observed a decrease in the α-NaGalase activity of the HuTu 80 and SK-MEL-28 cell lines after the treatment of the cells with CcPh. A similar effect was detected after the action of the brown algae fucoidan towards DLD-1 colorectal adenocarcinoma cells [20]. The marine sponge metabolites isoaaptamine, 9-demethylaaptamine, damiron B, and macaluvamine H also reduced the enzyme activity in the DLD-1 cell line from 100% to 64, 57, 52, and 50%, respectively, at a concentration of 5 µM [21]. However, neither fucoidan nor the marine sponge metabolites (the aaptamine and macaluvamine classes of alkaloids) directly inhibited α-NaGalase. Notably, the marine sponge-derived highly anti-cancer-active polybrominated diphenyl ethers [40,41] had no direct inhibitory effects on the cancer cell-associated α-NaGalase of the lines RPMI-7951 (ATCC #no. HTB-66™), MDA-MB-231 (ATCC#no.HTB-26™), DLD-1 (ATCC#no.CCL-221), HT-29 (ATCC#no.HTB-38™), HCT-116 (ATCC #no.CCL-247), and SK-MEL-28 (ATCC #no. HTB-72^TM^), as well as the murine healthy epidermal cell line JB6 Cl 41 (ATCC #no. CRL-2010™) [42]. At present, there is very little information concerning the inhibitors of GH27 family α-N-acetylgalactosaminidase. Clark et al. [43] showed that the iminosugar 2-acetamido-1,2-dideoxy-D-galactonojirimycin (DGJNAc) can inhibit, stabilize, and chaperone human α-NaGalase both in vitro and in vivo. Ayers et al [44] showed that galacto-iteamine, an unnatural analog of the alkaloid iteamine (o-aminobenzyl α-D-glucopyranoside) isolated from the sole plant *Itea virginica* L. inflorescence, was a weak direct inhibitor of α-NaGalase in chicken liver.

## 4. Materials and Methods

### 4.1. Materials and Reagents

Human duodenal carcinoma HuTu 80 (ATCC #no.HTB-40^TM^) and melanoma SK-MEL-28 (ATCC #no. HTB-72^TM^) cancer cells were obtained from the American Type Culture Collection (Manassas, VA, USA). Minimum Essential Medium (MEM) and Dulbecco’s Modified Eagle’s Medium (DMEM), phosphate-buffered saline (PBS), L-glutamine, penicillin–streptomycin solution, and trypsine were purchased from Sigma-Aldrich (St. Louis, MO, USA), while fetal bovine serum (FBS) was purchased from Biowest (Ranch, FL, USA), agar was purchased from Becton (Le Point-de-Claix, France), sodium hydrocarbonate (NaHCO_3_) was purchased from BioloT (St. Petersburg, Russia), p-nitrophenyl-N-acetyl-α-D-galactosaminide (p-NPNAGal) and Bradford reagent were purchased from Sigma-Aldrich (St. Louis, MO, USA), and recombinant protein markers for SDS-PAGE-electrophoresis were purchased from BioRad (1000 Alfred Nobel Drive, Hercules, CA, USA). The 3-(4,5-Dimethylthiazol-2-yl)-5-(3-carboxymethoxyphenyl)-2-(4-sulfophenyl)-2H-tetrazolium (MTS) samples were purchased from Promega (Fitchburg, WI, USA). Blotting Grade Bloker Non-Fat Dry Milk was purchased from BioRad (Hercules, CA, USA), primary NAGA Rabbit polyclonal antibody (PA5-97299) was purchased from Invitrogen (Rockford, IL, USA), Goat Anti-mouse IgG HRP-linked antibody was purchased from Sigma-Aldrich (St. Louis, MO, USA), and Anti-rabbit IgG and HRP-linked antibodies were purchased from Cell Signaling Technologies (Danvers, MA, USA). Polychrome-1 was purchased from Reakhim (Moscow, Russia), Sephadex^TM^ LH-20 was purchased from GE Healthcare (Bio-Sciences, Uppsala, Sweden), C-18 silica gel column was purchased from Sigma-Aldrich (St. Louis, MO, USA), and Sorbfil plates for TLC were purchased from ZAO Sorbopolimer (Krasnodar, Russia).

### 4.2. Experimental Equipment

Microplate spectrophotometer (BioTek Instruments, Highland Park, Winooski, VT, USA) was used for measuring optical density at 400 nm (D400). Ultrasonic homogenizer Bandelin Sonopuls (Bandelin electronic GmbH & Co., Berlin, Germany) was used for homogenization of cells’ biomass. GenBAflex-tubes 6–8 kDa (Scienova GmbH, Wildenbruchstabe, Jena, Germany) were used for dialysis. ^1^H NMR and ^13^C NMR spectra were recorded on a Bruker AVANCE DRX-500 NMR spectrometer at 500 and 125 MHz, respectively. Mini-PROTEAN Tetra Handcast Systems (Bio-Rad, Hercules, CA, USA) were used for SDS-PAG electrophoresis. GS-800 Calibrated Densitometr (Bio-Rad, Hercules, CA, USA) was used for gel visualization. Semi-dry transblot instrument from Bio-Rad (Hercules, CA, USA) and polyvinylidene difluoride membranes (PVDF) from Millipore (Billerica, MA, USA) were used for Western blot analysis. ChemiDoc M.D. Universal Hood III Gel Documentation System (Bio-Rad, Hercules, CA, USA) was used to visualize blots.

### 4.3. Brown Alga Phlorotannin Inverstigation

#### 4.3.1. Brown Alga Collection and Phlorotannins’ Isolation

The brown alga *C. costata* (Turn.) Saund (order Laminariales) was collected in Peter the Great Bay, Sea of Japan, in July 2020.

Phlorotannins were isolated from brown algae as described previously [35]. The fraction CcPh was characterized by nuclear magnetic resonance (NMR) spectroscopy and mass spectrometry as described below. Freshly collected brown alga *C. costata* (7000 g) was rinsed with fresh water, cleaned to remove epiphytes, dried with filter paper, crushed, and extracted with EtOH (96%, 10 L) for 30 days at room temperature. The extract was filtered, and an aliquot of 4.5 L was concentrated in vacuum to 1.3 L. The concentrate was extracted sequentially with hexane (3 × 1000 mL), CHCl_3_ (3 × 1000 mL), and EtOAc (3 × 1000 mL). The EtOAc extract was evaporated until reaching dryness. The obtained residue (1000 mg) was separated by chromatography using a column of silica gel (1500 × 150 mm) into 10 fractions (1−10) that were eluted sequentially by C_6_H_6_ (fraction 1) and C_6_H_6_–EtOAc (stepwise gradient, 10:1–1:1 (fractions 2–5), EtOAc (fraction 6) (409 mg), CHCl_3_ (fraction 7), CHCl_3_–EtOH 1:1 (fractions 8), and EtOH (fraction 9). Fraction 6 was separated over Polychrome-1 using H_2_O–EtOH (stepwise gradient) into five fractions (6.1–6.5). Fraction 6.3, eluted by H_2_O–EtOH (2.5:1, 133 mg), was separated over a column of silica gel 100C-18 using H_2_O–EtOH (stepwise gradient 0–96 in 5% steps) to fraction 6.3.20 (24.6 mg), which was eluted by EtOH (20%). Fraction 6.3.20 was separated over a column of sephadex LH-20 using 50% aqueous acetone into 4 fractions. Fraction 6.3.20.4 (10 mg), named CcPh, was used in the experiment.

Column fractions were analyzed by TLC on Sorbfil plates that were sprayed with FeCl_3_ solution (50%), followed by heating to 70 °C. R_f_ values were determined using Me_2_CO:C_6_H_6_:H_2_O:HCO_2_H (90:30:8:5 drops).

#### 4.3.2. Nuclear Magnetic Resonance Analysis

The NMR spectra of the CcPh fractions dissolved in DMSO-d were obtained on a Bruker Avance-III 500 HD spectrometer (Bruker, Karlsruhe, Germany) at an operating frequency of 500 MHz and at 35 °C with tetramethylsilane used as an internal standard.

#### 4.3.3. Mass Spectra Analysis

The high-resolution mass spectrometry (HRMS) data were collected using a hybrid ion trap–time-of-flight mass-spectrometer (LCMS-IT-TOF, Shimadzu, Japan). The mass spectra were recorded, applying negative ion electrospray ionization (ESI) mode with a resolution of 12,000. The following operating settings were used: the range of *m/z* detection was 300–2500, the drying gas (N_2_) pressure was 150 kPa, the nebulizer gas flow rate was 1.5 L/min, the ion source potential was 4.5 kV, the detector voltage was 1.65 kV, and the n temperatures for curved desolvation line (CDL) and heat block were 200 °C. The mass accuracy was below 4 ppm. Data were acquired and processed using the Shimadzu LCMS Solution software (v.3.60.361). Masses of phlorethols were calculated using the respective chemical formula of a typical phlorethol, C_6n_ H _4n+2_ O_3n_, where n is the number of phloroglucinol units.

### 4.4. Cell Culturing

Human duodenal carcinoma HuTu 80 and melanoma SK-MEL-28 cancer cells were grown in monolayer in Minimum Essential Medium (MEM) and Dulbecco’s Modified Eagle’s Medium (DMEM), respectively, with addition of 10% FBS and 1% penicillin-streptomycin solution. The cell cultures were maintained at 37 °C in humidified atmosphere containing 5% CO_2_

#### 4.4.1. Cytotoxic Activity Assays

Cancer cells (8 × 10^3^/200 µL) were seeded in 96-well plates for 24 h at 37 °C in a 5% CO_2_ incubator. The cells were treated with phlorethol CcPh at concentrations ranging from 0 to 200 µg for an additional 24 h. Subsequently, cells were incubated with 15 µL of MTS reagent for 3 h, and the absorbance in each well was measured at 490/630 nm using a microplate reader. All the experiments were repeated three times, and the mean absorbance values were calculated.

#### 4.4.2. Preparation of Cell Lysate

Every 3–4 days, HuTu 80 and SK-MEL-28 cells were rinsed in phosphate-buffered saline (PBS), detached from the tissue culture flask by 1X trypsin/EDTA solution, harvested with appropriate medium, and centrifuged at 500 rpm for 3 min. The culture media were discarded, and cell pellets were resuspended in 0.02% EDTA/15 mM Tris (pH 7.0) solution and frozen at −80 °C.

#### 4.4.3. Treatment of Cells by Phlorethol CcPh

HuTu 80 and SK-MEL-28 cells (5 × 10^5^ cells/dish) were seeded in 60 mm dishes. After 24 h, the cells were treated with a medium containing different concentrations of phlorethol CcPh (0, 10, 20, and 40 µg/mL). After 24 h, the cells were harvested, as described in Section 4.4.2., “Preparation of cells lysate”. After each treatment of the cells with phlorethol CcPh, cell lysates were prepared, the target enzyme was extracted, and its specific activity was determined, as described below in the Section 4.5.2.

### 4.5. Biochemical and Catalytic Properties of α-NaGalases

#### 4.5.1. Isolation and Purification of α-NaGalase from Cell Lysates

The frozen lysates of the cancer cells in the 15 mM Tris buffer, which were maintained at pH 7.0 and kept in 0.02% EDTA, were defrosted and sonicated 10 times at 20 s intervals with a break of 1 min in ice bath. To remove the cellular detritus, the cell homogenate was centrifuged at 4 °C and 10,000 rpm for 30 min. The supernatant proteins were precipitated with 70% ammonium sulfate and kept overnight at 4 °C to form a pellet. The protein pellet was collected by centrifugation (at 4 °C and 10,000 rev/min for 30 min) and dissolved in 0.05 M sodium citrate buffer at pH 5.0. The extract was dialyzed against the same buffer and centrifuged to separate the insoluble precipitate. The supernatant was used in further work as partly purified enzyme in the enzyme’s activity essays. The purification quality was controlled by 12% Laemmli-SDS-PAGE [45].

#### 4.5.2. Enzyme Assay

The activity α-NaGalase was determined by increasing the amount of p-nitrophenol (pNP). To assay the α-NaGalase activity, 10 µL of cell extract and 90 µL of substrate pNPNAGal (8.8 mM) in 0.05 M sodium citrate buffer, at pH 4.5, were placed in cells of 96-well plates and incubated at 37 °C for 5 h. The reactions were stopped by the addition of 200 µL of 1 M Na_2_CO_3_ solution. Absorbance of pNP was measured at 400 nm. Results were read with a computer program, Gen5, and processed with Excel software. The unit of standard activity (*U*) was defined as the amount of the enzyme catalyzing the formation of 1 nmol of pNP (ε_400_ = 18,300 M^−1^ cm^−1^) per 1 h under the conditions indicated by Formula (1):

(1) $$U=\frac{\Delta D\times V\times 1000}{18.3\times v\times \tau },$$

where ∆*D* = (*D*_400_ of the enzymatic reaction – *D*_400_ of the blank), *V* is the total volume of the reaction mixture (300 µL), *v* is the volume of the enzyme solution aliquot (10 µL), *τ* is the reaction time (h), and 1000 is the conversion factor in nmol.

Specific activity (*A*) was calculated as the standard enzyme activity per 1 mg of protein. All calculations were based on reactions with consumption of 10% of the chromogenic substrate. The protein concentrations were estimated by the Bradford method with BSA as a standard [46].

#### 4.5.3. pH Optimum of α-NaGalases Action

To determine the pH optimum of the enzyme, the mixture contained 10 µL of the enzyme solution (after the last stage of purification) and 90 µL of the substrate in a solution of 0.05 M Na–citrate buffer at pH 3.0–6.2 (initial concentration 3 mg/mL). Enzyme activity was determined after 5 h incubation at 37 °C, as described above.

#### 4.5.4. Catalytic Properties of α-NaGalases

To determine the *K*_m_ and *V*_max_ values of α-NaGgalase, a substrate solution of various concentrations was added to 10 μL of the enzyme solution (stock solution of protein was 1.2 and 0.8 mg/mL for HuTu 80 and SK-MEL-28 α-NaGalase, respectively) and incubated at 37 °C for 5 h. The final substrate concentrations in the incubation mixture were 0.07, 0.14, 0.28, 0.56, 1.13, 2.25, 4.50, and 9.0 mM. Reactions were stopped by adding 200 µL of Na_2_CO_3_. Activity was determined as described above. The Michaelis–Menten constants, *K*_m_ and *V*_max_, were determined from the non-linear regression coefficients using the Michaelis–Menten equation (Origin 8.1 software).

### 4.6. The Inhibitory Potency of the Phlorethol CcPh

For this experiment, 8 µL of enzyme (349 and 60 U/mL for HuTu 80 and SK-MEL-28 α-NaGalase, respectively) in 0.05 M sodium citrate buffer solution (pH 4.5) was preincubated for 30 min at 20 °C with 2 µL of water-soluble test compound at various concentrations (from 0 to 1.67 mg/mL in a probe) to facilitate enzyme and inhibitor’s interaction. The enzyme reaction was initiated by adding 90 µL of the substrate pNPNAGal (8.8 and 14 mM for HuTu 80 and SK-MEL-28 α-NaGalase, respectively) in the appropriate buffer solution. After 5 h, the reaction was stopped by adding 200 µL of 1 M Na_2_CO_3_. The amount of pNP was quantified by spectrophotometric detection at 400 nm. A mixture containing phlorethol CcPh at the appropriate concentration was used to compensate for the absorption at 400 nm by these compounds. Inhibition *I* (%), was calculated as follows (2):

(2) $$I=\frac{Ao-Ai}{Ao}\times 100$$

where *A*o is a specific activity of the enzyme in the absence of an inhibitor, calculated as described above. *A*i is the specific activity of the enzyme in presence of an inhibitor, calculated as follows (3):

(3) $$Ai=\frac{\left(D400\;reaction-D400\;blank-D400\;\mathrm{CcPh}\right)\times V\times 1000}{18.3\times v\times \tau \times mg\;of\;protein}\;,$$

where *D*_400 CcPh_ is the absorbance of CcPh at the appropriate concentration. 

The *IC*_50_ values were determined from the non-linear regression coefficients of the Hill’s equation using OriginLab software (version 8.1).

#### The Irreversibility of α-NaGalase Inhibition by Phlorethol CcPh

To determine the reversibility of the α-NaGalase inhibition by CcPh, 10 µL (10 mg/mL) of aqueous solution of phlorethol CcPh was added to 40 µL of the enzyme in 0.05 M sodium citrate buffer solution (pH 4.5). The remaining reaction mixture was dialyzed against 1 L of the 0.05 M sodium citrate buffer solution for 60 h at 4 °C. The buffer was changed 3 times during the dialysis. The enzymatic activity was determined as described above. A sample of α-NaGalase treated with H_2_O in the absence of the inhibitor was dialyzed and used as a control for determination of the initial activity. The experiment was carried out in two replicates. The degree of inhibition was calculated as above.

### 4.7. Relative Protein α-NaGalase Quantification in Lysates and Extracts of Cancer Cells by Western Blot Analysis

After HuTu 80 and SK-MEL-28 cells (6 × 10^5^) were cultured in a 7 cm dish overnight, they were treated with 10, 20, or 40 µg/mL of phlorethol CcPh for an additional 24 h. After treatment, the cells were harvested by 1X trypsin/EDTA solution and lysed with lysis buffer containing 0.88% NaCl, 50 mM Tris-HCl (pH 7.6), 1% NP-40, 0.25% NaClO_2_, 1 mM PMSF, and 1 mM Na_3_VO_4_, and then disrupted with sonication at 10 kHz 3 times for 10 s on ice. The suspension was centrifuged for 15 min at 13000 g and 4 °C. The resulting supernatant was used for Western blot analysis. Protein content was determined by Lowry assay [47]. Lysates were loaded onto 12% SDS-PAG and electrophoresed at a constant potential (100 V, 60 mA) in the discontinuous buffer system according to Laemmli-SDS-PAGE protocol. Separated proteins were electrophoretically transferred to polyvinylidene difluoride membranes (PVDF). The membranes were blocked with 5% non-fat milk for 1 h and then incubated with the respective specific primary antibody (β-actin and *α*-NaGalase at 1:1000 dilution) at 4 °C overnight. Protein bands were visualized using an enhanced chemiluminescence reagent (ECL Plus, GE Healthcare, Marlborough, MA, USA) after hybridization with Goat Anti-mouse IgG HRP-linked antibody (for b-actin) and Anti-rabbit IgG HRP-linked antibody (for *α*-NaGalase) (1:10,000 diluted). Band density was quantified via Quantity One 4.6 software (BioRad, Hercules, CA, USA).

### 4.8. Data Analysis

All figures shown in this study are representative of at least three independent experiments with similar results. Statistical differences were evaluated using the Student’s *t*-test and considered significant at * *p* < 0.05 and ** *p* < 0.01.

### 4.9. Molecular Docking of Phlorethol CcPh with Human α-NaGalase

The 3D-structures of the putative linear oligomers consisting of fifteen (P15OPh) or seven (PHPh) monomeric units (phloroglucinol) linked by aryl-ether bonds were built using the Molecule Build module of the Molecular Operating Environment package version 2020.09 (MOE, 2020.09; Chemical Computing Group ULC, Montreal, QC, Canada). The structures of P15OPh and PHPh were optimized using the Amber10:EHT forcefield. The crystal structure of human α-NaGalase (PDB ID 4DO4) [43] protonated at pH 5.0 was used for molecular docking via Docking module of the MOE 2020.09. Contact analysis of P15OPh was performed for a fragment smaller than 100 atoms (pentaphlorethol), since the Ligand Interaction module of the MOE program has a limitation for a ligand size of less than 100 atoms. The structures of 30 complexes were calculated with the London dG score, and the 5 most energetically advantageous complexes were optimized with the GBVI/WSA dG score. Contact analysis was carried out using the Ligand Interaction module of the MOE program.

## 5. Conclusions

The present study demonstrates the procedure for the purification of phlorotannin from the brown algae *C. costata* and its structural characterization by NMR spectroscopy and high-resolution mass spectrometry analysis. The phlorotannin profile contains several linear, inseparable compounds with many isomer forms and degrees of polymerization, from 11 to 23 phloroglucinol units, which bind with aryl–ether bonds, thereby revealing typical characteristics of phlorethols. The CcPh fraction decreased the activity of α-NaGalase in the HuTu 80 duodenal adenocarcinoma and SK-MEL-28 melanoma cell lines, and irreversibly inactivated the isolated enzymes. It was shown in silico that the oligophlorethols bind tightly to the active site of human lysosomal α-NaGalase. The terminal residues of oligophlorethols enter the active site and occupy the catalytic center between Asp 156 and Asp 217. This indicates that the inactivation of α-NaGalase by the CcPh inhibitor is directed to the active site. Thus, it can be concluded that the CcPh phlorethol fraction isolated from the brown algae *C. costata* is an effective marine-based natural inhibitor of cancer cell-associated, immunosuppressive α-NaGalase, which has immunomodulatory properties. This work constitutes an important contribution to understanding one of the aspects of the anticancer activity of this group of marine compounds. Based on these results, we suggest that phlorethol CcPh has high pharmaceutical and therapeutic potential.

## Figures and Tables

**Table 1:** Catalytic parameters *K*_m_ and *V*_max_ of α-NaGalases ^1^ from HuTu 80 and SK-MEL-28 cell lines.

Cell Lines	*K*_m_ (mM)	*V*_max_ (nmol/h/mL)
HuTu 80	4.20 ± 0.14	411.2 ± 6.5
SK-MEL-28	6.90 ± 0.43	138.8 ± 4.8

^1^ For p-NPNAGal as substrate in 0.05 M Na citrate buffer pH 4.5.

**Table 2:** HMBC * assignments of the CcPh fraction of *C. costata*.

Structure		^13^C (δ in ppm)	^1^H (δ in ppm)
Unsubstituted benzene carbons	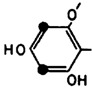	94.3	5.86–5.83
		93.8	5.94; 5.61
		94.5	6.16
Diaryl–ether bond (ether linkage)	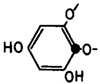	123.6	6.13–6.17
		123.5	5.93
		123.6	6.01
		123.5	5.59–5.62
		122.4	5.83–5.86
Benzene carbon bearing hydroxylated groups	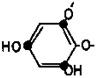	156.0	6.13–6.17
		153.9	5.59–5.62
		152.9	5.59–5.62
		150.9	6.13–6.17
		151.1	5.83–5.86
		154.0	5.83–5.86
		154.6	5.83–5.86

* CcPh were dissolved in DMSO-d with tetramethylsilane as the internal standard.

**Table 3:** The data concerning elemental compositions, monoisotopic masses, and ions of the CcPh *.

Degree of Polymerization	[M − 2H]^−2^		Signal Strength (%)	Elemental Composition	Monoisotopic Mass (Da)
	*m/z* Measured	*m/z* Calculated			
11	682.0898	682.0888	6	C_66_H_46_O_33_	1366.1921
12	744.0940	744.0968	12	C_72_H_50_O_36_	1490.2082
13	806.1019	806.1048	30	C_78_H_54_O_39_	1614.2242
14	868.1105	868.1129	46	C_84_H_58_O_42_	1738.2403
15	930.1183	930.1209	66	C_90_H_62_O_45_	1862.2563
16	992.1262	992.1289	99	C_96_H_66_O_48_	1986.2724
17	1054.1332	1054.1369	99	C_102_H_70_O_51_	2110.2884
18	1116.1430	1116.1449	74	C_108_H_74_O_54_	2234.3044
19	1178.1483	1178.1530	60	C_114_H_78_O_57_	2358.3205
20	1240.1588	1240.1610	50	C_120_H_82_O_60_	2482.3365
21	1302.1652	1302.1690	30	C_126_H_86_O_63_	2606.3526
22	1364.1746	1364.1770	16	C_132_H_90_O_66_	2730.3686
23	1426.1839	1426.1851	6	C_138_H_94_O_69_	2854.3847

* ESI–MS spectra were detected in negative ion mode HRMS at each degree of polymerization (DP), representing the predominant charge state detected under the experimental conditions described. The strength of signals expressed as a percentage (%) of intensity (100% = 550).

**Table 4:** Binding energy and hydrogen bonds of P15OPh fragment and DGJNAc with α-NaGalase.

P15OPh fragment								
Ligand			Receptor			Interaction	Distance	E (kcal/mol)
O	1	O	Pro	197	(A)	H-donor	2.66	−1.1
O	6	O	Glu	193	(A)	H-donor	2.63	−2.3
O	19	O	Gly	194	(A)	H-donor	2.65	−2.8
O	32	O	Ala	191	(A)	H-donor	2.79	−2.8
O	40	OD1	Asp	216	(A)	H-donor	2.54	−1.0
O	45	OD1	Asp	217	(A)	H-donor	2.57	−5.5
O	59	OD2	Asp	156	(A)	H-donor	2.52	−3.0
6-ring		CB	Leu	196	(A)	pi-H	4.31	−0.6
DGJNAc								
Ligand			Receptor			Interaction	Distance	E (kcal/mol)
N2	7	OD1	Asp	217	(A)	H-donor	2.81	−6.4
O4	17	OD1	Asp	78	(A)	H-donor	2.62	−3.4
O6	24	OD2	Asp	797	(A)	H-donor	2.73	−2.8
N5	26	OD2	Asp	156	(A)	H-donor	2.74	−17.3
C1	29	OD2	Asp	217	(A)	H-donor	3.55	−0.8
O7	1	OG	Ser	188	(A)	H-acceptor	2.63	−2.6
O3	13	NZ	Lys	154	(A)	H-acceptor	2.75	−7.0
O3	13	NH1	Arg	213	(A)	H-acceptor	3.07	−1.3
N5	26	OD2	Asp	156	(A)	ionic	2.74	-6.4

## Data Availability

Not applicable.
